# Synergizing Macrogeometric Design and Nano-Hydroxyapatite Coatings to Enhance Early Implant Stability and Bone Maturation

**DOI:** 10.3390/jfb17070316

**Published:** 2026-07-01

**Authors:** Ana Carolina Loyola Barbosa, Rafaella da Cruz Polizelli Scannavino, Uislen Berian Cadore, Arthur Belem Novaes Junior, Bruna Ghiraldini, Roberto Sales e Pessoa, Sergio Scombatti de Souza

**Affiliations:** 1 School of Dentistry of Ribeirão Preto, University of São Paulo, Ribeirão Preto 14040-904, SP, Brazil; anacarolinaloyolabarbosa@gmail.com (A.C.L.B.); rafapscannavino@usp.br (R.d.C.P.S.); uislenc@gmail.com (U.B.C.); novaesjr@forp.usp.br (A.B.N.J.); 2 Research & Development Department, S.I.N. Implant System, São Paulo 03033-021, SP, Brazil; bruna.ghiraldini@sinimplantsystem.com (B.G.); rp@inpes.com.br (R.S.e.P.); 3 Department of Periodontology and Implantology, School of Dentistry, University Centre of Triangulo-UNITRI, Uberlândia 38411-106, MG, Brazil

**Keywords:** dental implants, osseointegration, macrogeometry, surface treatment, micro-computed tomography, biomechanics

## Abstract

Implant macrogeometry and surface microstructure represent fundamental pillars for accelerating and enhancing the quality of osseointegration. The objective of this study was to evaluate the synergistic effect of three distinct implant designs, Strong (hybrid with trapezoidal threads), Unitite (hybrid with healing chambers), and Epikut (hybrid with active double threads), associated with two surface configurations (dual acid-etched [DAE] and nanohydroxyapatite [NanoHA]) on bone regeneration at 3 and 8 weeks. Twenty-four male rabbits randomly received forty-eight implants in their tibiae, yielding an equal accounting of *n* = 12 animals per experimental healing period (3 and 8 weeks) to ensure a balanced longitudinal and structural analysis. Biomechanical monitoring included final insertion torque (IT) and resonance frequency analysis (ISQ) at installation and euthanasia, while high-resolution micro-computed tomography (*µ*CT) quantified the 3D intersection surface index (IS/TS) and trabecular microarchitecture (BV/TV, Tb.Th, Tb.Sp, and Connectivity). Regarding insertion torque, no significant differences were observed between macrogeometries (*p* = 0.557), ensuring a standardized mechanical baseline for biological comparison. For clinical stability (ISQ), the Epikut and Unitite designs demonstrated significant stability gains as early as 3 weeks (*p* < 0.05). *µ*CT data confirmed a progressive, time-dependent structural reorganization, presenting a significant increase in trabecular thickness (Tb.Th) over time (*p* < 0.001), while the overall bone volume fraction (BV/TV, *p* = 0.861) and IS/TS index (*p* = 0.774) maintained statistical uniformity across all studied models. In conclusion, implant macrogeometry and surface nanotopography exert a distinct, chronologically shifted synergy during osseointegration. Clinically, these findings dictate target-specific selection: macrogeometric innovations accelerate early secondary clinical stabilization, whereas bioactive NanoHA coatings optimize subsequent long-term trabecular thickening within the established peri-implant architecture.

## 1. Introduction

The discovery of functional anchorage between living bone and loaded titanium implants, termed osseointegration, has revolutionized patient rehabilitation over recent decades [1,2]. This dynamic process begins with immediate mechanical fixation, known as primary stability, which is gradually replaced by secondary stability through biological events involving healing, resorption, and new bone formation [3,4]. In clinical practice, the transition between these phases is critical; thus, measuring stability through insertion torque (IT) and the implant stability quotient (ISQ) via resonance frequency analysis has become essential to ensure treatment predictability and enable early loading protocols [5,6].

Primary stability is influenced by a complex interplay of factors, including the bone quality of the recipient site and, predominantly, the device’s macrostructure [7]. Variables such as diameter, length, taper, and thread architecture determine the degree of compression and the initial mechanical interlocking within the cortical bone [8]. Although the patient’s bone density is often an intrinsic biological limitation, the refinement of macrostructural design and the customization of the surgical preparation represent the primary pathways for intervention to optimize the initial biomechanical performance of implants [9].

To maximize bone-to-implant contact (BIC) and accelerate integration, novel macrogeometric concepts have been introduced, including hybrid designs and modifications in thread cutting efficiency [10]. A significant innovation was the creation of “healing chambers”, spaces between threads designed to stabilize the blood clot and favor cellular recruitment without direct compression of the prepared bone tissue [8]. Pre-clinical studies indicate that geometries incorporating these chambers, particularly those with trapezoidal profiles, can result in superior bone deposition and a reduction in the time required for effective integration [11,12].

Simultaneously with macroscopic innovations, modifications in surface microstructure play a vital role in accelerating the interfacial biological response. While dual acid-etching (DAE) is widely utilized to create a microroughness that favors early osseointegration, its efficacy depends strictly on the chemical protocol employed [13,14]. Currently, coating surfaces with nanohydroxyapatite (NanoHA) emerges as a promising alternative due to its similarity to the mineral phase of bone, being capable of inducing the phenomenon of biointegration and promoting more accelerated bone healing compared to surfaces purely conditioned by acid [15,16].

Despite the documented benefits of both advanced macrogeometries and nanosynthetic surfaces, the scientific literature lacks evidence regarding the synergistic effect between these two factors. Previous investigations by our group confirmed the superiority of NanoHA surfaces in systemically compromised models; however, these studies utilized mini-implants with a single geometry, which prevents understanding how different commercial thread designs and diameters influence these outcomes [17,18]. Consequently, a significant knowledge gap persists concerning the intricate interplay between macrogeometric design and surface microstructure in governing the transition from mechanical to biological stability, a process that warrants further elucidation through high-resolution, three-dimensional analytical frameworks.

This investigation is justified by the need to fill this gap using an experimental model that utilizes implants with clinically relevant dimensions and designs. The use of micro-computed tomography (*µ*CT) allows for an analysis of the peri-implant trabecular microarchitecture in 3D, providing volumetric and structural data that complement clinical IT and RFA findings. Understanding the isolated and combined impact of each modification is fundamental to enhancing clinical predictability, allowing for the development of systems that ensure accelerated stability even in challenging scenarios.

Considering the above, the objective of this study was to evaluate the synergistic effect of three distinct implant designs, hybrid with trapezoidal threads, conical with healing chambers, and hybrid with active threads, associated with DAE or NanoHA surfaces; through the analysis of insertion torque, stability quotients (ISQ), and 3D microtomographic parameters (BV/TV, Tb.Th, Tb.Sp). We hypothesized that the combination of chamber-based or prominent thread designs with bioactive nanohydroxyapatite coatings significantly accelerates early osseointegration and enhances peri-implant trabecular microarchitecture compared to conventional micro-rough surfaces.

## 2. Materials and Methods

### 2.1. Ethical Committee Submission

The present project was submitted to and approved by the Ethics Committee on the Use of Animals (CEUA) of the School of Dentistry of Ribeirão Preto, University of São Paulo (FORP/USP)—CEUA Protocol: 0047/2022. The procedures followed the ethical norms governed by the Brazilian College of Animal Experimentation (COBEA).

### 2.2. Sample Size Calculation

The sample size estimation was performed using the G*Power software (version 3.1.9.7; Heinrich Heine University Düsseldorf), aiming to ensure a statistical power of 80% with a significance level *α* of 0.05. The calculation parameters were based on bone-to-implant contact (BIC) data reported by Cohen et al. (2016), considering the variation in means (17.93%) and the standard deviation (1.69%) observed [19]. Based on these criteria, the use of nine specimens per experimental period was defined. Based on these criteria, the use of nine specimens per experimental period was defined. Sample size was powered for differences in trabecular and osseointegration outcomes, using the variability from BIC as a proxy.

### 2.3. Sample Characterization

Twenty-four healthy, adult, male New Zealand rabbits were used, weighing approximately 2.5 kg to 3.2 kg and aged 6 months. Only male rabbits were selected to eliminate potential confounding effects of cyclic estral hormonal variations on bone remodeling and osseointegration rates. The animals were randomly assigned to the six experimental groups using a computer-generated randomization sequence to minimize selection bias (detailed in the Supplementary Material, Table S1). Throughout the entire experimental period, including the post-operative phase, the rabbits were housed individually in separate appropriate cages (1 cage per rabbit) to prevent aggressive behavior or surgical site accidents. Animals remained in the Vivarium of the School of Dentistry of Ribeirão Preto, FORP-USP, under controlled environmental conditions with a 12 h light cycle and temperature between 22 and 24 °C, with food and water ad libitum. All subsequent volumetric and statistical evaluations were conducted by investigators blinded to the experimental group identities to ensure objective data acquisition.

### 2.4. Implant Installation

The animals were previously weighed for the calculation of the anesthetic dose and post-operative medication. General anesthesia was obtained by the association of Ketamine Hydrochloride (Agener União Ltda, São Paulo, SP, Brazil) and Xylazine Hydrochloride (Rompum; Bayer SA, São Paulo, SP, Brazil), at doses of 50 mg/kg and 5 mg/kg respectively, via intramuscular route. Additionally, the anti-inflammatory Meloxicam at 0.2% (Ouro Fino, Cajamar, SP, Brazil) was administered at a dose of 1 mg/kg via subcutaneous route. Subsequently, trichotomy and asepsis with 10% alcoholic PVPI were performed.

The implant installation surgery was performed as an adaptation of the methodology proposed by Cohen et al. [19] in a study which concluded that the position of implant installation in the rabbit tibia influences the quality of osseointegration. Thus, the implants were positioned in two positions, A and B: position A at 5 mm distance from the joint between the tibia and the femur; position B at 5 mm from position A. In this sense, randomization of the position of the implants by group/surface/position in the tibiae was performed, ensuring that each experimental group had an equivalent number of implants in each position in the tibia at the same evaluation time, so that the installation position would not influence the results.

On the medial face, in its most voluminous portion of bone tissue, an incision was made with a number 3 scalpel handle and 15C scalpel blade (Swann-Morton, Sheffield, England) in the skin, along the long axis of the tibiae. The muscular tissue was sectioned and set aside until a clear visualization of the periosteum was achieved. The periosteum was then detached with the help of a Molt detacher. Next, the preparation of the surgical sites to receive the implants was performed under abundant and continuous irrigation with sterile saline solution, strictly adhering to the instrumentation sequences recommended by the manufacturer (S.I.N.—Implant System, São Paulo, Brazil) for each specific system. The osteotomies were prepared utilizing the initial and pilot drills at a rotational speed of 1200 rpm, followed by the subsequent shaping drills at a standardized speed of 800 rpm to carefully control bone preparation, minimize thermal allocation, and optimize subsequent thread engagement. Following site preparation, all implants were mechanically installed with the assistance of a low-rotation surgical motor (ExpertSurg, Nobel Biocare, Gothenburg, Sweden) at 20 rpm. Implants were driven without countersinking until the implant platform was perfectly coincident and flush with the external cortical plate of the tibia.

**Figure 1:**
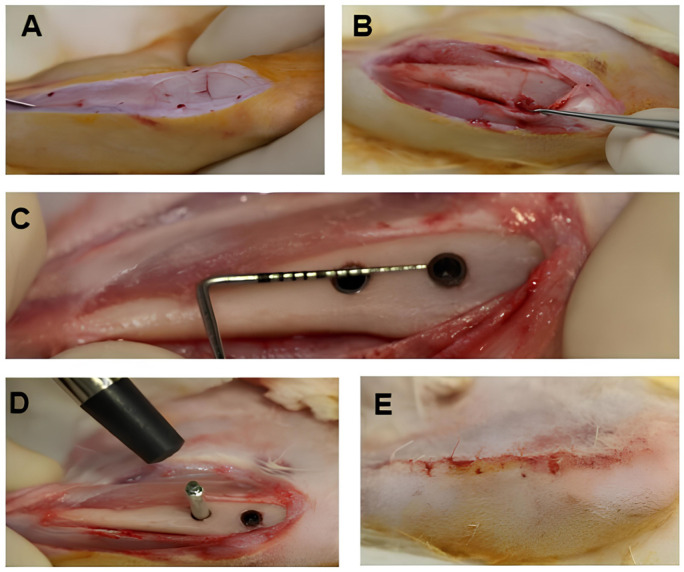
Sequence of the surgical procedure for implant installation in rabbit tibia. (**A**) Linear incision on the medial face of the tibia and exposure of the periosteum. (**B**) Linear reference on the cortical surface for osteotomy planning; the starting point (position A) is located 5 mm from the femorotibial joint, serving as a base for the future demarcation of position B, positioned 5 mm distally to this initial point. (**C**) Installation of the implants respecting the inter-implant distance of 5 mm. (**D**) Measurement of primary stability (ISQ) through the connection of SmartPegs and reading by resonance frequency. (**E**) Tissue synthesis by planes with interrupted sutures using 5.0 Vicryl absorbable suture thread.

Two implants were installed in each tibia of the rabbit, 5.0 mm away from each other. For the positioning of the implants in each animal, a randomization plan was followed. For each implant installed, the final torque indicated on the motor was recorded as the Insertion Torque for that implant. The motor used (ExpertSurg, Nobel Biocare, Gothenburg, Sweden) has a resolution of 1 Ncm for torque measurement. After that, an appropriate SmartPeg was connected to each implant, and the reading of the Resonance Frequency value—ISQ was performed using the Osstell (Osstell, Gothenburg, Sweden), being recorded as the installation ISQ. The operator performing the torque and ISQ measurements was fully blinded to the implant surface treatments to prevent evaluation bias. After the readings, the SmartPegs were disassembled. Next, the surgery was finalized with sutures in planes with simple stitches using absorbable suture threads (Vicryl Ethicon 5.0, Johnson Prod., São José dos Campos, Brazil). The surgical procedure was performed as shown in Figure 1.

The animals received post-operative antibiotic medication (single dose) via intramuscular route of 24,000 IU/kg of Penicillin G-benzathine at a dose of 0.01 mL for each 100 g of the rabbit’s body weight (Pentabiótico Veterinário Pequeno Porte, Fort Dodge^®^ Saúde Animal Ltda., Campinas, SP, Brazil) and analgesia with 2% Tramadol Hydrochloride (Cronidor^®^- União Química Farmacêutica Nacional S/A, Embu-Guaçu, SP, Brazil) at a dosage of 3 mg/kg via subcutaneous route. Tramadol hydrochloride (3 mg/kg) for analgesia and Meloxicam (anti-inflammatory) were administered subcutaneously every 12 and 24 h post-operatively, respectively. After the surgical procedure, the animals were kept in appropriate cages, without restriction of movement or feeding throughout the experimental period.

### 2.5. Characterization of the Implants

All implants installed are off-the-shelf implants from the company S.I.N.—Implant System, São Paulo, with Morse taper prosthetic connection and dimensions of 3.5 mm in diameter by 8.5 mm in length, differing in macrogeometry and surface treatment, commercially available under the names Strong SW^®^, Unitite^®^, and Epikut^®^. The macrogeometry and thread format of the implants are described in Table 1.

The three models of implant macrostructures were used both with dual acid-etched (DAE) surfaces, with nitric acid and sulfuric acid, and with nanohydroxyapatite (NanoHA) surfaces. Thus, there were 3 macrostructures and 2 surfaces evaluated. The characterization of these surfaces was addressed previously by Oliveira et al. (2020) and will be briefly described in the following two paragraphs [17].

The implants were produced from commercially pure titanium cylindrical bars (grade 4), according to the diameter and length characteristics provided for in the project, in CNC (Computer Numeric Control) lathes, by a machining process. Subsequently, they received automated pre-washing by Centrifugal Disc units, and a hygiene process, performed in controlled rooms (Clean Room), in high-performance automated cleaning systems (ultrasonic cleaning systems). The DAE surface was obtained from a machined implant surface that received baths of nitric acid followed by sulfuric acid, in a micro-corrosion process [17].

To obtain the NanoHA surface treatment, a DAE implant surface was processed [1]; briefly, the coating liquid containing nanohydroxyapatite crystals was applied to the top of the implant to be coated, and the implant was placed in a “spin coater” device. The implant was rotated at 2600 rpm for 3 s for homogenization of the liquid throughout the surface and allowed to dry for 10 min at room temperature. The implant was then placed in an oven at 450 °C for 5 min for sintering and stable adhesion of the nanohydroxyapatite crystals [17].

### 2.6. Experimental Groups

The specimens were strategically divided into six experimental groups, each containing nine implants per euthanasia interval, totaling 24 animals (12 per period). The groups were defined as follows:

- Strong DAE Group: Strong SW implants with dual acid-etched surface treatment;
- Strong NanoHA Group: Strong SW implants with nanohydroxyapatite surface treatment;
- Unitite DAE Group: Unitite implants with dual acid-etched surface treatment;
- Unitite NanoHA Group: Unitite implants with nanohydroxyapatite surface treatment;
- Epikut DAE Group: Epikut implants with dual acid-etched surface treatment;
- Epikut NanoHA Group: Epikut implants with nanohydroxyapatite surface treatment.

The complete randomization matrix, detailing the distribution between right and left tibiae and anatomical positions (A or B), is provided in the Supplementary Material (Table S1).

### 2.7. Insertion Torque Analysis

For each implant installed, the final torque indicated on the motor was recorded as the Final Insertion Torque of that implant. The motor used (ExpertSurg, Nobel Biocare, Gothenburg, Sweden) had a resolution of 1 Ncm for the measurement of insertion torque. Mean insertion torques were then calculated by experimental group, and comparative statistical analyses were performed between the groups.

### 2.8. Euthanasia Procedure

After 3- and 8-week periods, the animals were euthanized by anesthetic overdose associated with the inhalation of CO_2_ in an appropriate environment. The tibiae were promptly collected and reduced into blocks containing the devices, using precision diamond discs (KG Sorensen, São Paulo, Brazil) coupled to a handpiece. Next, the fragments were washed in PBS solution and stored in 10% buffered formalin solution. This fixation protocol aimed to preserve the integrity of the bone-implant interface for performing micro-computed tomography (*µ*CT).

### 2.9. Resonance Frequency Analysis

Implant stability was monitored by means of Resonance Frequency Analysis (RFA) in two chronological stages: immediately after implant installation and at the time of euthanasia of the animals. The protocol consisted of coupling a magnetic transducer SmartPeg™ (Osstell, Gothenburg, Sweden) compatible with the prosthetic platform of each device. Readings were obtained with the aid of the Osstell device (Osstell, Gothenburg, Sweden), whose tip was brought close to the upper end of the SmartPeg™ without physical contact (Figure 1). A quadruple measurement per implant was carried out, with 90° variations between the probe positions. The final stability value (ISQ) for each experimental period corresponded to the arithmetic mean resulting from the four measurements carried out after validation by the equipment’s sound signal.

### 2.10. Microtomographic Analysis (µCT)

The bone-implant specimens were fixed in 10% buffered formalin, with an initial solution replacement performed within the first 48 h to ensure effective stabilization. Subsequently, the fixative was replaced weekly over a 5-week period, ensuring optimal tissue preservation and maintaining structural integrity for high-resolution micro-computed tomography (*µ*CT) analysis. Images were captured in a SkyScan 1172-160 microtomograph (Bruker, Kontich, Antwerp, Belgium) operating at 100 kV and 100 μA. To optimize image quality, an Al-Cu filter was used, with a pixel size of 5.87 μm and 360° rotation (step of 0.40°). The processing of the raw images was performed in the NRecon software (version 1.7.4.2; Bruker), while the three-dimensional orientation and selection of the sagittal axis of interest occurred in the DataViewer software (version 1.5.6.2; Bruker).

The quantification of bone parameters was performed in the CTAn software (v.1.15.4.0; Bruker, Kontich, Belgium) through advanced processing algorithms. The delimitation of the Region of Interest (ROI) included the total length of the implant, starting 1 mm distal to its coronal platform. To specifically evaluate the peri-implant bone response, the Volume of Interest (VOI) was defined as the bone tissue located within the implant threads, extending from the implant surface to the edge of the thread tips. The morphometric parameters analyzed within this VOI included: relative bone volume (BV/TV, %), trabecular thickness (Tb.Th, mm), trabecular separation (Tb.Sp, mm), trabecular number (Tb.N, 1/mm), and connectivity (Conn, %). Density levels for binarization were fixed at 35–150 for bone and 150–255 for the implant. To ensure methodological rigor, all *µ*CT evaluations were performed by a single operator, blinded to the experimental group identities.

To confirm the reliability and reproducibility of the microtomographic measurements, an intra-operator consistency assessment was performed prior to the formal analysis. The single blinded operator repeated the region of interest (ROI) and volume of interest (VOI) selection on 10 randomly selected samples with a two-week interval, demonstrating an intraclass correlation coefficient (ICC) greater than 0.90 for all morphometric parameters.

### 2.11. Statistical Analysis

All statistical procedures were performed by a trained investigator blinded to the experimental group identities. Prior to comparative analysis, data normality and homoscedasticity were verified using the Shapiro–Wilk and Brown–Forsythe tests, respectively. For the clinical metrics of initial mechanical stability, including IT and primary ISQ, a two-way Analysis of Variance (ANOVA) was utilized. The three-dimensional micro-computed tomography (*µ*CT) parameters were evaluated using a three-way Analysis of Variance, operationalized via a General Linear Model (GLM), to assess the isolated effects of macrogeometry, surface treatment, and healing time, as well as their potential synergistic interactions. In all instances where global analysis or factor interactions achieved statistical significance, data were further explored using Tukey’s post hoc test for multiple comparisons. For all statistical decisions, the significance level was fixed at 5% (*p* < 0.05). Statistical processing and modeling were conducted using SigmaPlot software (version 14.5; Systat Software Inc., San Jose, CA, USA), while graphical representations and trend illustrations were prepared using Microsoft Excel (Microsoft 365, version 2402; Microsoft Corporation, Redmond, WA, USA).

## 3. Results

During the reporting period, all planned surgical procedures were successfully completed, including the installation of 96 implants in 24 rabbits and the execution of the two euthanasia stages (at 21 and 56 days). Following sacrifice, the specimens were retrieved in block and fixed in 10% buffered formalin for subsequent processing. The surgical phase proceeded as planned without complications. Animal adaptation was satisfactory, and the anesthetic and analgesic management protocol proved effective. The randomization process ensured a homogeneous distribution of the different implant models and surface treatments between the right and left sides of the tibiae.

### 3.1. Insertion Torque and Resonance Frequency Analysis

**Figure 2:**
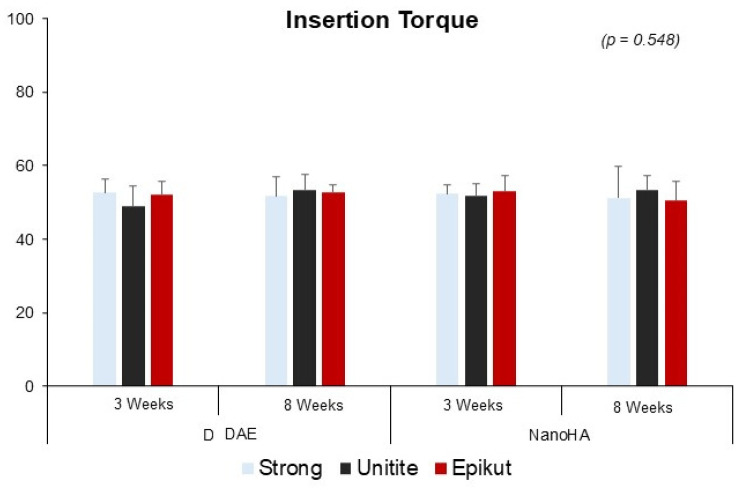
Insertion Torque: Insertion Torque values (Ncm) according to implant macrogeometry and surface treatment. Data presented as Mean and Standard Deviation, confirming baseline homogeneity (*p* > 0.05). Regarding stability measured by resonance frequency analysis (ISQ), a statistically significant increase was detected between the initial period and the time of euthanasia (*p* < 0.001). This gain in clinical stability was significantly observed as early as the 3-week interval for the Epikut DAE, Epikut NanoHA, and Strong DAE groups (*p* < 0.05). When analyzing progression by macrostructure, it was observed that the Unitite^®^ and Epikut^®^ designs reached high secondary stability levels earlier, whereas the Strong system showed a gradual gain, reaching ISQ values comparable to the other groups by the 8-week period.

Initial clinical analysis revealed that all implant systems achieved adequate primary stability levels at the time of installation. The mean insertion torque (Ncm) and respective standard deviations for the experimental groups were: Strong DAE (52.68 ± 3.65), Strong NanoHA (52.37 ± 2.50), Unitite DAE (48.98 ± 5.57), Unitite NanoHA (51.91 ± 3.04), Epikut DAE (52.18 ± 3.67), and Epikut NanoHA (53.00 ± 4.14). According to the Analysis of Variance (ANOVA), no statistically significant differences were observed in insertion torque regarding macrogeometry (*p* = 0.557) or surface (*p* = 0.548), as illustrated in Figure 2. These data ratify that the initial mechanical stability was homogeneous among all groups, regardless of the tested design or surface treatment.

**Figure 3:**
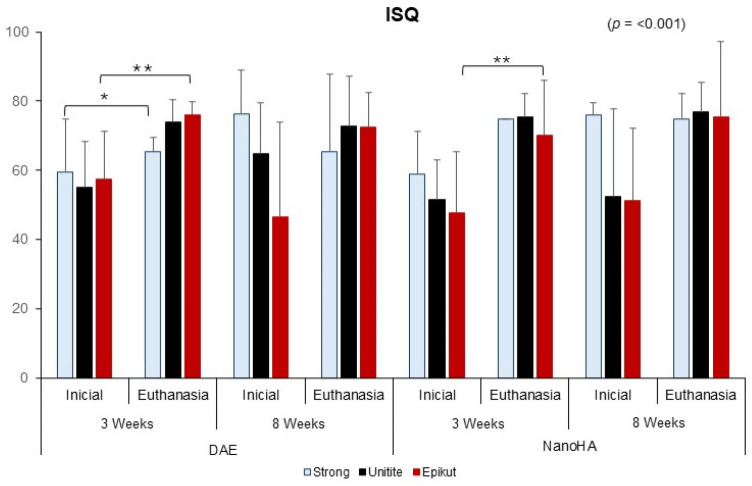
Implant Stability Quotient (ISQ) measured immediately after insertion (Baseline) and at the time of euthanasia (Final). Brackets indicate statistically significant increases in stability over time (intra-group comparison) for specific groups in the 3-week interval (*p* < 0.01). At the 8-week evaluation, no significant differences were observed between the different macrogeometries. The asterisks indicate statistically significant differences (* *p* < 0.05, ** *p* < 0.01).

Regarding the volumetric interaction, DAE and NanoHA surface treatments demonstrated similar behaviors across all implant designs, with no isolated statistical differences between them (*p* > 0.05), indicating that both surfaces were effective in promoting the transition from mechanical to biological stability. Overall, the mean ISQ value progressed from 59.6 at installation to 76.0 at the end of the experiment. ANOVA identified no significant differences for insertion torque between macrogeometries (*p* = 0.557) or surfaces (*p* = 0.548), confirming the homogeneity of the primary stability obtained (Figure 3), while ISQ data ratified the positive evolution of clinical stability throughout the healing time.

### 3.2. Micro-Computed Tomography (µCT) Parameters

Qualitative analysis through three-dimensional reconstructions revealed bone neoformation in direct contact with the surface of all evaluated implants, regardless of experimental group or observation period. In all processed samples, the absence of fibrous tissue interposition at the bone-implant interface was verified, indicating the progression of the osseointegration process at both 3 and 8 weeks. Images demonstrated that the neoformed bone tissue presented continuity with the remaining bone of the tibial metaphyseal region, filling the surroundings of the implants throughout their perimeter.

**Figure 4:**
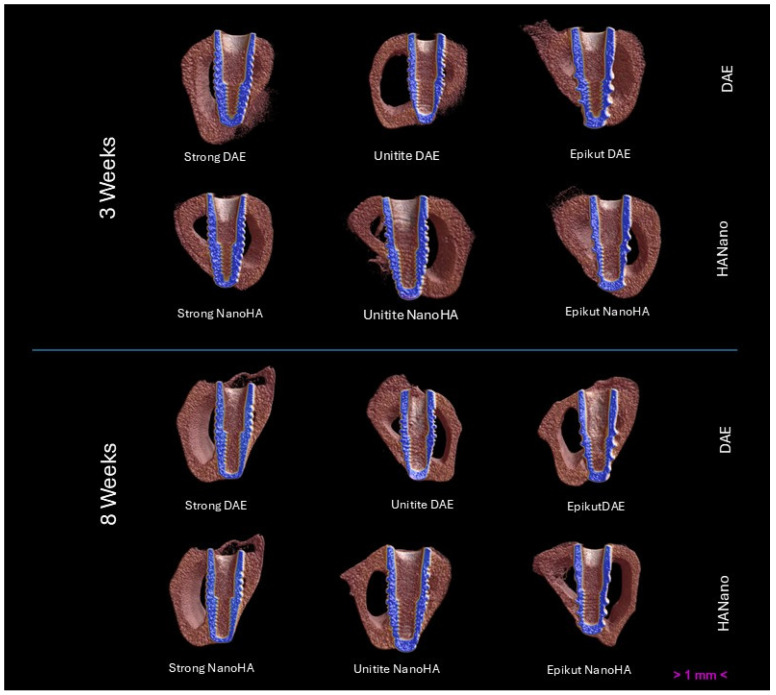
Three-dimensional reconstructions obtained by micro-computed tomography (*µ*CT) representative of the six experimental groups after 3 and 8 weeks of healing. Neoformed and remaining bone tissue is represented in brown, while implants are represented in blue, allowing for an illustration of the volumetric interaction and peri-implant trabecular architecture in each experimental group.

As presented in Figure 4, distinct patterns of tissue distribution conditioned by the macrogeometry of each system were observed. In hybrid implants with more aggressive threads (Strong and Epikut groups), bone deposition was predominantly concentrated in the spaces delimited by the implant threads. Conversely, in the Unitite^®^ groups, bone filling occurred characteristically within the healing chambers, where a well-distributed and organized trabecular network was observed since the initial 3-week period. Specifically for the Unitite^®^ groups, this morphological perception was confirmed by quantitative parameters, which demonstrated a significant increase in relative bone volume (BV/TV) from 28.31% to 32.18% (*p* < 0.001) and in trabecular thickness (Tb.Th) from 0.23 mm to 0.27 mm (*p* < 0.001) between the 3- and 8-week periods, reflecting efficient bone maturation within the chambers.

**Figure 5:**
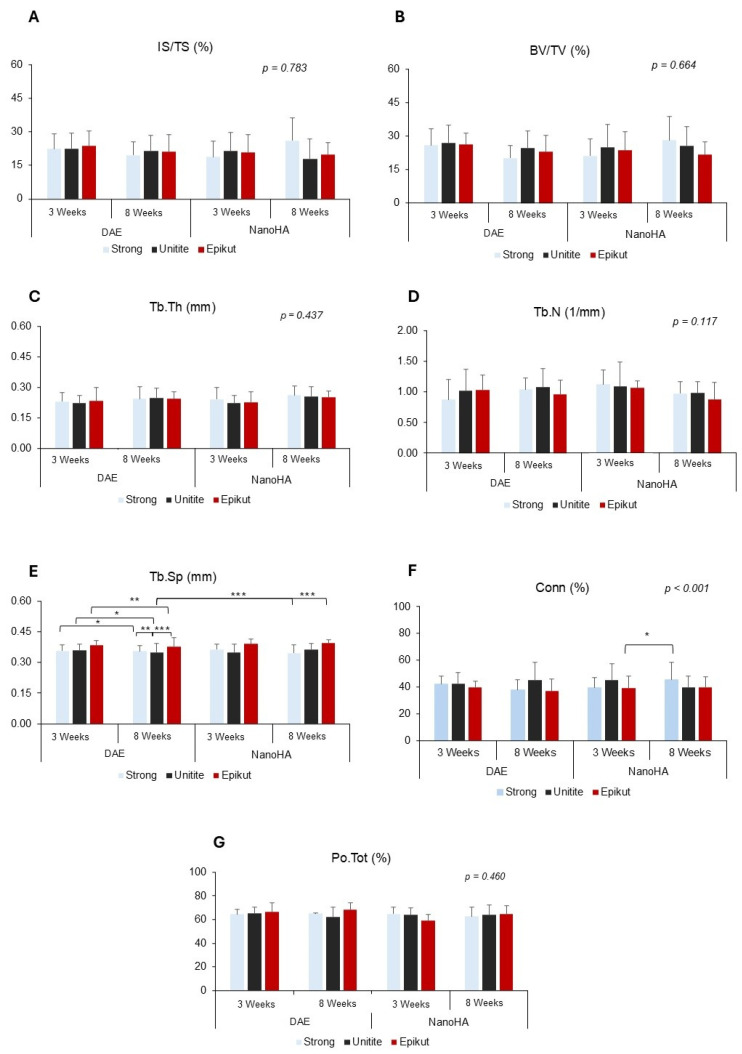
Microtomographic evaluation of bone microarchitecture parameters highlighting temporal maturation. (**A**) IS/TS, (**B**) BV/TV, (**C**) Trabecular Thickness (Tb.Th); (**D**) Trabecular Number (Tb.N); (**E**) Trabecular Separation (Tb.Sp); (**F**) Connectivity (Conn); (**G**) Total Porosity (Po.Tot). Brackets indicate statistically significant differences between the 3- and 8-week experimental periods and between the groups evaluated in the final period. The level of statistical significance is represented by the number of asterisks: * (*p* < 0.05); ** (*p* < 0.01); *** (*p* ≤ 0.001). Data expressed as Mean and Standard Deviation, analyzed by ANOVA with Tukey’s post-test.

**Figure 6:**
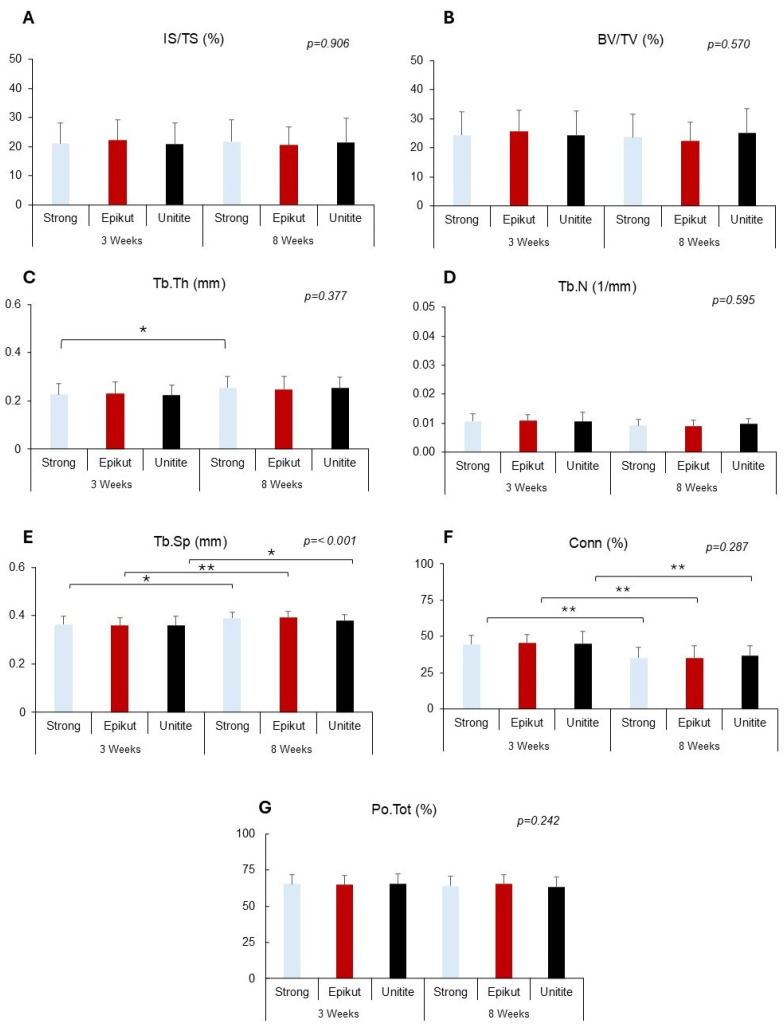
Quantitative microtomographic analysis of peri-implant bone microarchitecture and volumetric parameters. (**A**) Intersection Surface (IS/TS); (**B**) Bone Volume Fraction (BV/TV); (**C**) Trabecular Thickness (Tb.Th); (**D**) Trabecular Number (Tb.N); (**E**) Trabecular Separation (Tb.Sp); (**F**) Connectivity (Conn); and (**G**) Total Porosity (Po.Tot). Brackets and asterisks indicate statistically significant differences between the 3- and 8-week experimental periods and between specific macrogeometries. The level of significance is represented by: * (*p* < 0.05) and ** (*p* < 0.01). Data are expressed as Mean ± Standard Deviation. Statistical analysis was performed using two-way ANOVA followed by Tukey’s *post hoc* test.

**Figure 7:**
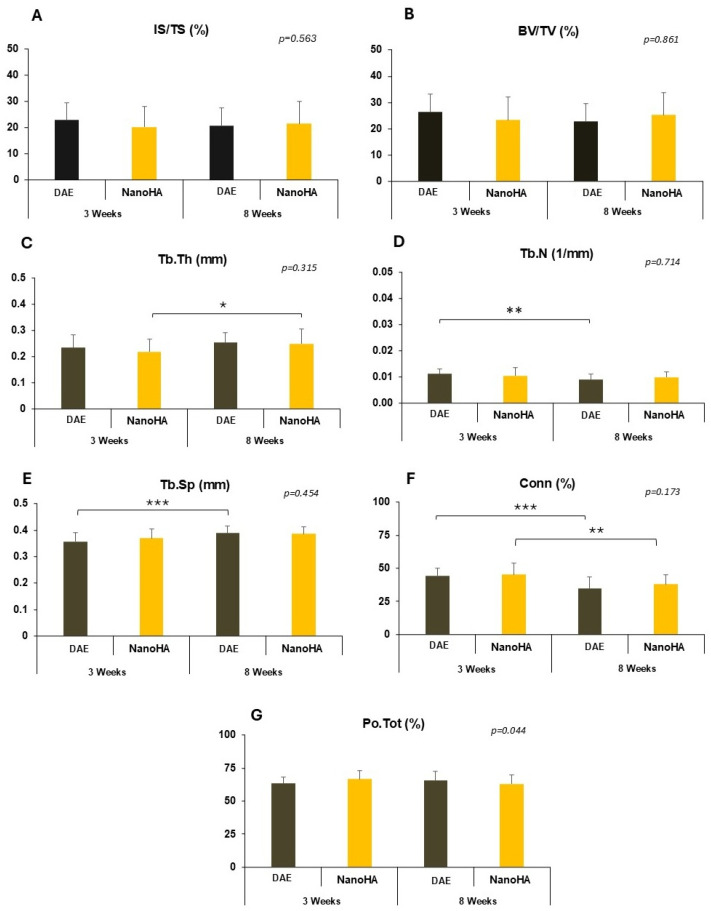
Comparative analysis of the effect of surface treatment over time. Comparison between dual acid-etched (DAE) and nanohydroxyapatite (NanoHA) surfaces at the 3- and 8-week periods. The graphs present the parameters of: (**A**) Intersection Surface (IS/TS); (**B**) Bone Volume Fraction (BV/TV); (**C**) Trabecular Thickness (Tb.Th); (**D**) Trabecular Number (Tb.N); (**E**) Trabecular Separation (Tb.Sp); (**F**) Connectivity (Conn) and (**G**) Total Porosity (Po.Tot). Brackets with asterisks indicate statistically significant differences in the temporal evolution of each isolated surface (*p* < 0.05). The asterisks indicate the levels of statistical significance: * *p* < 0.05, ** *p* < 0.01, and *** *p* ≤ 0.001.

**Figure 8:**
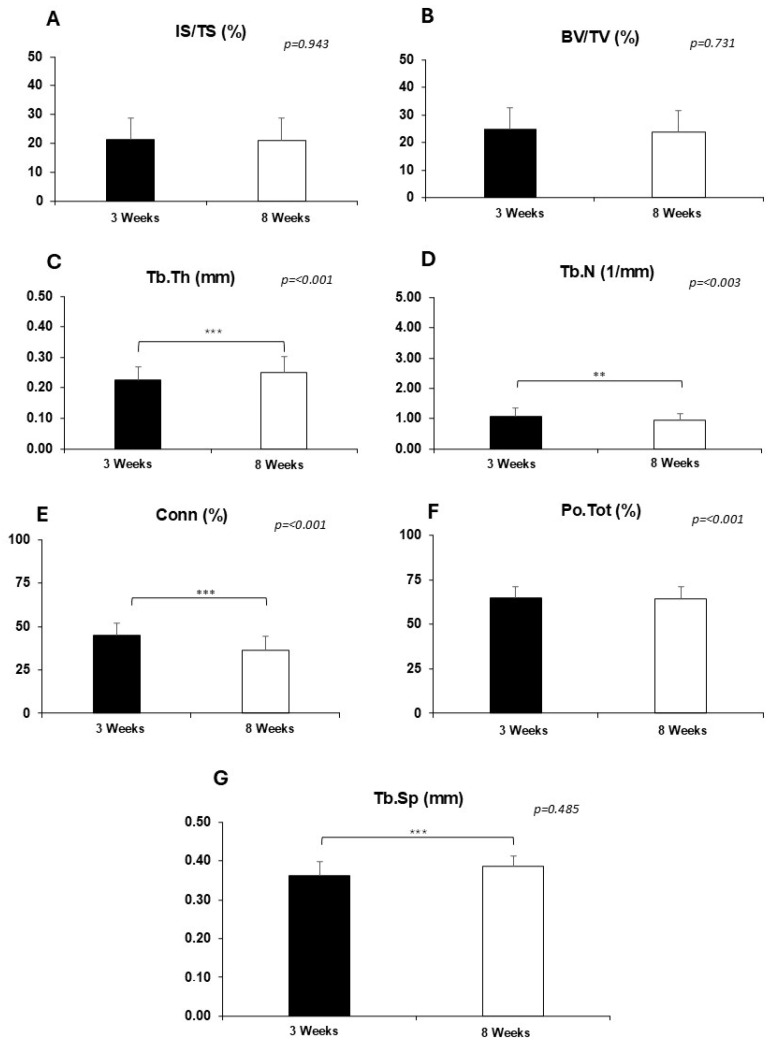
Global evaluation of temporal bone maturation between 3 and 8 weeks. Comparative analysis of bone morphometric parameters highlighting the primary effect of healing time, regardless of implant macrogeometry or surface treatment. The longitudinal evolution of peri-implant bone microarchitecture is presented through: (**A**) Intersection Surface (IS/TS); (**B**) Bone Volume Fraction (BV/TV); (**C**) Trabecular Thickness (Tb.Th); (**D**) Trabecular Number (Tb.N); (**E**) Trabecular Separation (Tb.Sp); (**F**) Connectivity (Conn); and (**G**) Total Porosity (Po.Tot). Asterisks indicate statistical significance levels: ** *p* < 0.01; *** *p*< 0.001. Data represents the pooled mean ± standard deviation for each time point.

The quantitative evaluation, illustrated through the longitudinal data in Figure 5, Figure 6, Figure 7 and Figure 8, revealed that healing time was the primary determining factor for the structural maturation of the peri-implant bone. In a global analysis of the pooled data (Figure 5), while volumetric parameters remained relatively stable, the trabecular microarchitecture underwent a significant reorganization characterized by a generalized increase in trabecular thickness and a concomitant reduction in connectivity, regardless of the implant design or surface treatment. Regarding volumetric interaction, the intersection surface index (IS/TS) and bone volume fraction (BV/TV) manifested tissue uniformity around all devices throughout the study. For the IS/TS index, the stability of mean values between 3 weeks (21.53 ± 7.19%) and 8 weeks (21.14 ± 7.65%) confirmed the absence of a main effect for time (*p* = 0.774), surface (*p* = 0.563), or macrogeometry (*p* = 0.906). Similarly, the overall BV/TV presented means of 24.83 ± 7.86% in the initial period and 23.92 ± 7.81% in the final stage, not being significantly influenced by surface type (*p* = 0.861), macrogeometry (*p* = 0.570), or healing period (*p* = 0.606), as shown in Figure 6A,B and Figure 7A,B.

In contrast to the statistical stability of the total bone volume fraction, the peri-implant trabecular microarchitecture underwent a significant structural reorganization over time. For Tb.Th, a significant main effect of time was detected (*p* < 0.001, Figure 5C), resulting in superior means at 8 weeks (0.27 ± 0.01 mm) compared to 3 weeks (0.23 ± 0.01 mm). Specifically, when evaluating factors in isolation, the NanoHA surface presented a significant increase in thickness at 8 weeks (*p* = 0.033, Figure 7C), a trend also observed for the Strong macrogeometry (*p* = 0.027, Figure 6C). In contrast, trabecular connectivity (Conn) significantly reduced with bone maturation (*p* < 0.008, Figure 5F). Connectivity was superior in the initial period for all implant models (*p* < 0.006) and both surface treatments (*p* < 0.002), as evidenced in Figure 6F, Figure 7F and Figure 8F. Regarding trabecular number (Tb.N), data did not demonstrate statistically relevant variations (*p* = 0.345, Figure 6D), indicating that numerical density remained stable while trabeculae became thicker and less connected.

The parameter Trabecular spacing (Tb.Sp) was significantly influenced by both time and macrogeometry (*p* < 0.001, Figure 6E). The increase in Tb.Sp between 3 and 8 weeks was significant for Epikut (*p* = 0.005), Strong (*p* = 0.049), and Unitite (*p* = 0.027) implants, reflecting bone reorganization. Tukey’s post hoc test identified that the Unitite DAE group at 8 weeks (0.36 ± 0.04 mm) presented significantly lower spacing compared to Strong DAE (0.40 ± 0.03 mm; *p* < 0.001), Epikut NanoHA (0.40 ± 0.02 mm; *p* < 0.001), Epikut DAE (0.39 ± 0.04 mm; *p* = 0.001), and Strong NanoHA (0.38 ± 0.04 mm; *p* = 0.010). Regarding total porosity (Po.Tot), no significant differences were found between macrogeometries (*p* = 0.242, Figure 6G). However, a statistical interaction between surface and time was detected (*p* = 0.044, Figure 7G), indicating that surface behavior regarding porosity was dependent on the healing period, although subsequent paired comparisons did not isolate specific differences within each evaluated time point.

## 4. Discussion

The paradigm of osseointegration has evolved from purely morphological descriptions toward a sophisticated functional and biomechanical investigation of the bone-implant interface [20]. The findings of this investigation suggest that implant macrogeometry and surface topography do not function as isolated variables; rather, they exert a synergistic influence that modulates both the kinetics of clinical stabilization and the architectural quality of peri-implant bone [5].

Regarding initial stability, the absence of statistically significant differences in insertion torque values across various macrogeometries (*p* = 0.557) constitutes a pivotal methodological finding. This data suggests that the surgical instrumentation and site preparation protocol effectively standardized the mechanical interlocking, neutralizing inherent thread design variations at the moment of placement [21]. Consequently, all experimental groups initiated from a comparable mechanical baseline, a condition that validates subsequent biological comparisons [22]. Nevertheless, primary stability must be recognized merely as the nascent stage of a dynamic biological process, which is significantly dictated by three-dimensional thread architecture [23].

The transition toward biological stability, as measured by ISQ, unveiled distinct patterns among the evaluated systems. The Epikut DAE, Epikut NanoHA, and Strong DAE groups exhibited significant stability increments as early as the three-week interval (*p* < 0.05). As proposed by Coelho et al. [24], designs that prioritize blood clot preservation and ensure a balanced mechanical strain may expedite stability recovery following the critical initial healing phase. In the specific case of the Unitite^®^ system, the integration of healing chambers provides protective niches that mitigate stress on nascent tissue, potentially fostering more predictable and accelerated osteoconduction [15,24].

The volumetric analysis (microtomographic BV/TV parameter) underscored a notable trend toward the superiority of the Unitite^®^ system, which demonstrated high levels of cortical bone filling regardless of the surface treatment employed. The biological rationale for this observation aligns with the healing chamber theory [25]. Designs that maintain voids during insertion allow for simultaneous bone formation from both the osteotomy walls and the implant surface [8]. This sequestered environment facilitates early bone deposition, yielding a filling volume that typically exceeds designs characterized by higher tissue compression [21,26].

The significant increase in relative bone volume (BV/TV) observed between three and eight weeks (*p* < 0.001), which progressed from a global mean of 28.31% to 32.18%, points to a non-linear stability trajectory. This phenomenon indicates that osseointegration followed a biological maturation trajectory, where neoformed tissue evolved into a higher degree of structural organization [27]. This volumetric expansion likely reflects the transition from woven bone to organized lamellar bone, suggesting that the tested designs did not impede natural bone consolidation kinetics [28].

Concerning trabecular microarchitecture, trabecular thickness (Tb.Th) also demonstrated a statistically significant increase over time (*p* < 0.001), rising from 0.23 mm to 0.27 mm. This structural reinforcement indicates a healthy remodeling process where trabeculae thicken to accommodate future biomechanical demands [29]. While stability metrics (ISQ) track the velocity of the interfacial union, trabecular bone volume (*µ*CT) appears to be primarily regulated by the regional host biology and the systemic health of the recipient [17].

The role of surface topography in modulating trabecular microarchitecture must be analyzed, as the superior performance of the NanoHA treatment in enhancing bone thickness (*p* = 0.033) suggests a significant influence of biochemical interlocking. While conventional DAE surfaces rely primarily on microroughness for initial cellular adhesion, the deposition of nanohydroxyapatite crystals points toward a biomimetic adaptation of the mineral phase of bone tissue. Previous investigations suggest that this nanometric configuration may facilitate the expression of non-collagenous proteins, such as osteocalcin, at earlier stages than those observed on purely micro-rough surfaces [15,30]. This mechanism suggests that, although the implant macrogeometry determines the available space, the surface nanostructure may regulate the mineralization rate of the newly formed bone.

Regarding the isolated influence of macrostructures, the behavior of groups with prominent thread profiles (Strong^®^ and Epikut^®^) during the initial three-week period warrants analysis. The stability increments observed in these designs (*p* < 0.05) point to the role of mechanical friction generated by the thread blades within the cortical bone. However, the stability of the trabecular number (Tb.N) index across all groups suggests that the compressive stress inherent in these designs did not reach levels that compromised regional biological viability. This finding points to the fact that the adopted surgical protocol allowed for an equilibrium between primary fixation and the preservation of trabecular numerical density, avoiding bone resorption processes secondary to excessive pressure often reported in high-compression designs [31]. Standardizing this mechanical baseline is crucial when translating preclinical data into immediate loading protocols, where preventing micro-motion during the critical transition to secondary stability dictates long-term implant survival, as demonstrated by Pantaleo et al. [32] in long-term jaw rehabilitations.

When evaluating the distinctive performance of the Unitite^®^ system, the presence of lower trabecular spacing (Tb.Sp, *p* < 0.01) compared to the other groups suggests an impact of chamber-based designs on bone architecture. Unlike hybrid profiles that prioritize immediate mechanical anchoring, healing chambers may favor intramembranous-like bone formation within protected niches [26]. The literature points out that low hydrostatic pressure environments within concavities suggest a facilitation of early angiogenesis and vascular influx [26,31]. From a translational perspective, fostering a tighter trabecular mesh and maximizing early bone filling within these chambers plays a major role in preserving crestal bone architecture and maintaining long-term marginal bone stability under dynamic functional loading, aligning with the multicenter evidence presented by Trombelli et al. [33] (2024) regarding peripheral factors affecting peri-implant resorption over time. Therefore, the denser trabecular organization observed in these groups suggests that clot protection and the sequestered space act as factors that support the volumetric superiority identified in the qualitative analyses.

Despite these insights, certain limitations warrant consideration. The rabbit tibia model, although a gold standard for surface evaluation, exhibits a more accelerated bone metabolism than humans, requiring careful extrapolation of the data to clinical practice [19]. Moreover, the lack of functional loading in this model restricts the understanding of these macrogeometries under dynamic stress. Future research incorporating compromised models such as those involving Diabetes Mellitus [18] or environmental factors like smoke exposure [34] may reveal even more pronounced interactions among the variables analyzed.

In summary, the integration of 3D morphometric and biomechanical data suggests distinct clinical profiles for each technology. The divergence between the speed of clinical stabilization (led by Unitite^®^ and Epikut^®^) and the progressive trabecular maturation is a notable finding.

Extrapolating the present results to a clinical scenario, the Unitite^®^ system emerges as a viable option when early volumetric filling is paramount, while the Epikut^®^ NanoHA system offers advantages in situations requiring accelerated stabilization. These hypotheses speculated here need to be tested in controlled and randomized clinical trials in humans. Ultimately, osseointegration success depends on the judicious selection of a system that aligns with the specific biological exigencies of each patient.

## 5. Conclusions

Within the limits of this preclinical investigation, it can be concluded that implant macrogeometry and surface nanotopography exert a distinct, chronologically shifted synergy rather than a simple additive effect on peri-implant healing kinetics. Macrogeometric innovations, specifically healing chambers (Unitite^®^) and active double threads (Epikut^®^), promote significantly faster gains in secondary clinical stability (ISQ) within the first 3 weeks of healing, securing the physical space and mechanical baseline required during critical early transition phases. Concurrently, the overall bone volume fraction (BV/TV) remains stable across variations, while the underlying trabecular microarchitecture undergoes a healthy, time-dependent physiological remodeling. This maturation is characterized by a progressive increase in trabecular thickness (Tb.Th)—which is selectively modulated and enhanced at the later healing stage by the bioactive NanoHA coating—alongside increased trabecular spacing (Tb.Sp) and reduced connectivity (Conn). Ultimately, different implant systems demonstrate distinct biomechanical and structural profiles, suggesting that while specific designs favor accelerated clinical stabilization, the final quality of the mineralized trabecular network is maintained, and the choice of system should be guided by the specific biological requirements of the clinical site.

## Figures and Tables

**Table 1:** Description of the macrogeometry and thread format of the implants used in the study.

Implants	Macrogeometry	Thread Format
Strong SW^®^	Hybrid (cylindrical and conical)	Trapezoidal
Unitite^®^	Hybrid (cylindrical and conical)	Internal and external threads (Healing Chamber)
Epikut^®^	Hybrid (cylindrical and conical)	Double Cutting threads

## Data Availability

The data presented in this study is available on request from the corresponding author.

## Supplementary Materials

The following supporting information can be downloaded at: https://www.mdpi.com/article/10.3390/jfb17070316/s1, Table S1: Randomized distribution of the experimental groups to be followed, with randomization of the right and left tibiae and the position of the implants (A or B). For the 3 and 8-week period.

## Abbreviations

The following abbreviations are used in this manuscript:

*µ*CT	Micro-Computed Tomography
ISQ	Implant Stability Quotient
IT	Insertion Torque
DAE	Dual Acid-Etching
CEUA	Ethics Committee on the Use of Animals
COBEA	Brazilian College of Animal Experimentation
NanoHA	Nanohydroxyapatite
BV/TV	Bone Volume/Total Volume
Tb.Th	Trabecular Thickness
Tb.Sp	Trabecular Spacing
Conn	Connectivity
RFA	Resonance Frequency Analysis
VOI	3D Volume of Interest
ROI	Region of Interest
