# Supplementary material for: Synergizing Macrogeometric Design and Nano-Hydroxyapatite Coatings to Enhance Early Implant Stability and Bone Maturation
Source: J Funct Biomater. 2026 Jul 1;17(7):316. doi: 10.3390/jfb17070316 (PMC13412758; doi:10.3390/jfb17070316)
Supplement: Supplementary file 1 [file jfb-17-00316-s001.zip › jfb-4317835-supplementary.pdf]

# Supplementary Materials

# Synergizing Macrogeometric Design and Nano-Hydroxyapatite Coatings to Enhance Early Implant Stability and Bone Maturation

**Ana Carolina Loyola Barbosa [1,2], Rafaella da Cruz Polizelli Scannavino [1], Uislen Berian Cadore [1,2], Arthur Belem Novaes, Jr. [1], Bruna Ghiraldini [2], Roberto Sales e Pessoa [2,3] and Sergio Scombatti de Souza [1,*]**

[1] School of Dentistry of Ribeirão Preto, University of São Paulo, Ribeirão Preto 14040-904, SP, Brazil; anacarolinaloyolabarbosa@gmail.com (A.C.L.B.); rafapscannavino@usp.br (R.d.C.P.S.); uislenc@gmail.com (U.B.C.); novaesjr@forp.usp.br (A.B.N.J.)

[2] Research & Development Department, S.I.N. Implant System, São Paulo 03033-021, SP, Brazil; bruna.ghiraldini@sinimplantsystem.com (B.G.); rp@inpes.com.br (R.S.e.P.)

[3] Department of Periodontology and Implantology, School of Dentistry, University Centre of Triangulo-UNITRI, Uberlândia 38411-106, MG, Brazil

* Correspondence: e-mail: scombatti@forp.usp.br

**Table S1**: Randomized distribution of the experimental groups to be followed, with randomization of the right and left tibiae and the position of the implants (A or B). For the 3 and 8-week period.

| Animal | Period | Right Tibia (Pos. A) | Right Tibia (Pos. B) | Left Tibia (Pos. A) | Left Tibia (Pos. B) |
|---|---|---|---|---|---|
| 01 | 3 Weeks | Strong NanoHA | Epikut DAE | Strong DAE | Unitite NanoHA |
| 02 | 3 Weeks | Epikut DAE | Strong DAE | Epikut NanoHA | Unitite DAE |
| 03 | 3 Weeks | Strong NanoHA | Epikut NanoHA | Unitite NanoHA | Epikut DAE |
| 04 | 3 Weeks | Strong DAE | Strong NanoHA | Epikut DAE | Epikut NanoHA |
| 05 | 3 Weeks | Epikut NanoHA | Unitite NanoHA | Epikut DAA | Strong DAA |
| 06 | 3 Weeks | Strong NanoHA | Epikut NanoHA | Unitite DAA | Unitite NanoHA |
| 16 | 3 Weeks | Strong DAA | Unitite DAA | Unitite NanoHA | Epikut DAA |
| 08 | 3 Weeks | Epikut NanoHA | Strong NanoHA | Epikut DAA | Strong DAA |
| 09 | 3 Weeks | Strong NanoHA | Epikut NanoHA | Strong DAA | Unitite DAA |
| 10 | 3 Weeks | Epikut DAA | Strong NanoHA | Unitite NanoHA | Unitite DAA |
| 11 | 3 Weeks | Unitite NanoHA | Epikut DAA | Unitite DAA | Strong NanoHA |
| 12 | 3 Weeks | Unitite DAA | Strong DAA | Epikut NanoHA | Unitite NanoHA |
| 01 | 8 Weeks | Strong NanoHA | Epikut DAA | Strong DAA | Unitite NanoHA |
| 02 | 8 Weeks | Epikut DAA | Strong DAA | Epikut NanoHA | Unitite DAA |
| 17 | 8 Weeks | Strong NanoHA | Epikut NanoHA | Unitite NanoHA | Epikut DAA |
| 15 | 8 Weeks | Strong DAA | Strong NanoHA | Epikut DAA | Epikut NanoHA |
| 13 | 8 Weeks | Epikut NanoHA | Unitite NanoHA | Epikut DAA | Strong DAA |
| 14 | 8 Weeks | Strong NanoHA | Epikut NanoHA | Unitite DAA | Unitite NanoHA |
| 07 | 8 Weeks | Strong DAA | Unitite DAA | Unitite NanoHA | Epikut DAA |
| 18 | 8 Weeks | Epikut NanoHA | Strong NanoHA | Epikut DAA | Strong DAA |
| 09 | 8 Weeks | Strong NanoHA | Epikut NanoHA | Strong DAA | Unitite DAA |
| 19 | 8 Weeks | Epikut DAA | Strong NanoHA | Unitite NanoHA | Unitite DAA |
| 20 | 8 Weeks | Unitite NanoHA | Epikut DAA | Unitite DAA | Strong NanoHA |
| 21 | 8 Weeks | Unitite DAA | Strong DAA | Epikut NanoHA | Unitite NanoHA |
